# Mind-Body Exercise Modulates Locus Coeruleus and Ventral Tegmental Area Functional Connectivity in Individuals With Mild Cognitive Impairment

**DOI:** 10.3389/fnagi.2021.646807

**Published:** 2021-06-14

**Authors:** Jiao Liu, Jing Tao, Rui Xia, Moyi Li, Maomao Huang, Shuzhen Li, Xiangli Chen, Georgia Wilson, Joe Park, Guohua Zheng, Lidian Chen, Jian Kong

**Affiliations:** ^1^College of Rehabilitation Medicine, Fujian University of Traditional Chinese Medicine, Fuzhou, China; ^2^National-Local Joint Engineering Research Center of Rehabilitation Medicine Technology, Fujian University of Traditional Chinese Medicine, Fuzhou, China; ^3^Department of Psychiatry, Massachusetts General Hospital and Harvard Medical School, Charlestown, MA, United States; ^4^Traditional Chinese Medicine Rehabilitation Research Center of State Administration of Traditional Chinese Medicine, Fujian University of Traditional Chinese Medicine, Fuzhou, China; ^5^Key Laboratory of Orthopedics & Traumatology of Traditional Chinese Medicine and Rehabilitation, Fujian University of Traditional Chinese Medicine, Ministry of Education, Fuzhou, China; ^6^Department of Rehabilitation Psychology and Special Education, University of Wisconsin, Madison, WI, United States; ^7^School of Nursing and Health Management, Shanghai University of Medicine and Health Sciences, Shanghai, China

**Keywords:** Baduanjin, resting state functional connectivity, mild cognitive impairment, locus coeruleus, ventral tegmental area

## Abstract

Mild cognitive impairment (MCI) is a common global health problem. Recently, the potential of mind-body intervention for MCI has drawn the interest of investigators. This study aims to comparatively explore the modulation effect of Baduanjin, a popular mind-body exercise, and physical exercise on the cognitive function, as well as the norepinephrine and dopamine systems using the resting state functional connectivity (rsFC) method in patients with MCI. 69 patients were randomized to the Baduanjin, brisk walking, or healthy education control group for 6 months. The Montreal Cognitive Assessment (MoCA) and magnetic resonance imaging (MRI) scans were applied at baseline and at the end of the experiment. Results showed that (1) compared to the brisk walking, the Baduanjin significantly increased MoCA scores; (2) Baduanjin significantly increased the right locus coeruleus (LC) and left ventral tegmental area (VTA) rsFC with the right insula and right amygdala compared to that of the control group; and the right anterior cingulate cortex (ACC) compared to that of the brisk walking group; (3) the increased right LC-right insula rsFC and right LC-right ACC rsFC were significantly associated with the corresponding MoCA score after 6-months of intervention; (4) both exercise groups experienced an increased effective connectivity from the right ACC to the left VTA compared to the control group; and (5) Baduanjin group experienced an increase in gray matter volume in the right ACC compared to the control group. Our results suggest that Baduanjin can significantly modulate intrinsic functional connectivity and the influence of the norepinephrine (LC) and dopamine (VTA) systems. These findings may shed light on the mechanisms of mind-body intervention and aid the development of new treatments for MCI.

## Introduction

Mild cognitive impairment (MCI) is a condition characterized by impaired cognitive function with a minimal impact on activities of daily living. Current pharmacologic treatments for the condition are unsatisfactory. Exercise has been recently recommended to improve cognitive function in patients with MCI (Petersen et al., 2018). Literature also suggests that mindful movement (mind-body intervention), which combines mindfulness and physical exercise, may have synergistic effects and lead to better outcomes than those achieved from either physical exercise or mindfulness meditation alone (Burgener et al., 2008).

Baduanjin is a mind-body exercise that focuses on both mindfulness and the strengthening of muscles and tendons (Liao et al., 2015). Previous studies have found that Baduanjin can improve attention, executive control function, and memory function, as well as modulate cognition-related brain function and structure. For instance, we found that compared to the education control, 12-week Baduanjin training significantly improved the memory quotient (MQ) and resting state functional connectivity of dorsal lateral prefrontal cortex (DLPFC) and hippocampus in elderly adults (Tao et al., 2016, 2017a). In another study in individuals with MCI, we found compared to the brisk walking and control groups, the Baduanjin can increased memory function as measured by Montreal Cognitive Assessment (MoCA), modulate the brain low-frequency oscillations, and increase gray matter volume of hippocampus and anterior cingulate cortex (ACC) (Tao et al., 2019).

More recently, a systematic review and meta-analysis of randomized controlled trials from 1054 participants showed that compared with conventional therapy, Baduanjin plus conventional therapy significantly improved cognitive and memory function in patients with mild cognitive impairment (Yu L. et al., 2020). In another pilot study, Xiao et al. (2020) found that community-delivered Baduanjin training program was safe for prefrail/frail older adults with the potential to improve cognitive function measured by MoCA.

Although accumulating evidence has demonstrated the potential of Baduanjin for cognition/memory improvement, its underlying mechanism remain unclear. Literature suggests that the release of stress-induced catecholamines may play an important role in cognitive processes (Zhang et al., 2016). Animal studies have found that the locus coeruleus (LC), a brain region involved in many cognitive processes, is a major node of norepinephrine (NE) release in the stress response (Liu et al., 2017). Takahashi et al. showed that compare to the healthy control, the LC contrast ratios significantly reduced in patient with Alzheimer’s disease and mild cognitive impairment (Takahashi et al., 2014). Lee et al found that older adults were associated with decline in LC functional connectivity with frontoparietal networks that coordinate attentional selectivity (Lee et al., 2018).

Dopamine is another important catecholamine and a neurotransmitter involved in the reward and motivation process. Literature suggests that the ventral tegmental area (VTA), which projects to the nucleus accumbens and ACC (Navratilova et al., 2015), plays a crucial role in the release of dopamine (Lammel et al., 2014). Accumulating literature suggest that dopamine is involved in both dementia (Koch et al., 2020) and exercise (Fan et al., 2021). For instance, a previous study found that degeneration of VTA dopaminergic neurons at pre-plaque stages contributes to memory deficits and dysfunction of reward processing in dementia (Nobili et al., 2017).

Locus coeruleus-NE and VTA-dopamine systems have many similarities in physiological effects, and both are responsive to motivationally salient events (such as reward predictors) (Ranjbar-Slamloo and Fazlali, 2020). Disturbances of both have been implicated in highly overlapping sets of clinical disorders, such as attention deficit disorder (Unsworth and Robison, 2017) and Autism (Huang et al., 2021). The interaction of these two systems may plan an important role in pathophysiology of cognitive disorders such as MCI (Aston-Jones and Cohen, 2005).

In the present study, we comparatively investigated the modulation effects of 6 months of Baduanjin exercise, compared to brisk walking and a healthy education control, on resting state functional connectivity (rsFC) of key regions of the norepinephrine (LC) and dopamine (VTA) systems. In addition, we also applied exploratory effective connectivity analysis and region-of-interest-based gray matter volume (GMV) analysis in key regions derived from the LC and VTA rsFC analysis. We hypothesized that, compared to the brisk walking and healthy education control interventions, Baduanjin exercise would significantly modulate the LC and VTA resting state functional connectivity and effective connectivity within key regions of the two systems (amygdala, prefrontal cortex, hippocampus/parahippocampus for LC (Zhang et al., 2016; Jacobs et al., 2018)) and mesolimbic and mesocortical pathways such as the ACC, medial prefrontal cortex, and hippocampus for VTA (Zhang et al., 2016; Yu S. et al., 2020). Additionally, we hypothesize that the functional connectivity changes may be associated with the cognitive function improvement.

## Materials and Methods

The randomized controlled trial with three parallel groups (Baduanjin, brisk walking, and a healthy education control) was approved by the Medical Ethics Committee of the Second People’s Hospital of Fujian Province (Fuzhou, China) and was registered in the Chinese Clinical Trial Registry (ChiCTR-ICR-15005795). All subjects gave their informed consent at the initiation of study procedures, and details of these procedures can be found in our previous publication (Tao et al., 2019) in which we investigate the modulation effect of Baduanjin using the Amplitude of low-frequency fluctuations (ALFF), region-of-interest voxel-based morphometry (VBM) Analysis, and rsFC of hippocampus and ACC (based on the ALFF results). In this study, we focused on the modulation effect of Baduanjin on the LC-NE and VTA-dopamine system, which has not been previously published. Approximately half of all subjects each group who enrolled in the clinical trial were randomly selected to undergo MRI/functional MRI brain scans (*n* = 23 each group). This manuscript focuses on the participants with MRI data.

### Patients

Inclusion criteria included: 1. Aged 60 years or older; 2. No regular physical exercise for at least half a year (exercise with a frequency of at least twice a week and 20 min per session); 3. Having a physician’s diagnosis of MCI based on the Petersen diagnostic criteria (Petersen, 2004); 4. Memory problems with MoCA score < 26 (if years of education ≤ 12, one point will be added to the patient’s score); 5. Cognitive decline in accordance with age and education; 6. Intact activities of daily living (Lawton–Brody ADL score < 18); 7. Absence of dementia (Global Deterioration Scale score at 2 or 3).

Subjects were excluded from the study if any of the following criteria were met: 1. Resistant hypertension; 2. Severe vision or hearing loss; 3. Evidence of severe psychiatric conditions (such as active suicidal ideation, schizophrenia, etc.) or Geriatric depression scale score (GDS) ≥ 10 (Scale, 1997); 4. Current severe medical conditions for which exercise is contraindicated; 5. History of alcohol or drug abuse; 6. Participation in other clinical studies; 7. Pharmacologic treatments that may interfere with cognitive function.

### Intervention

All participants in the study received health education every 8 weeks for 30 min per session. In these sessions, participants were educated on ways to prevent the development of MCI.

Participants in the Baduanjin and brisk walking groups received the 24-week exercise training with a frequency of 3 days/week, 60 min/day (15-min warm up, 40-min training, and 5-min cool down). Research staff members also participated in all training sessions to record the attendance of each subject.

Baduanjin training was based on the ‘Health Qigong Baduanjin Standard,’ which was enacted by the Chinese State Sports General Administration in Health Qigong Management Center of General Administration of Sport of China. (2003) and consists of 10 postures (including the preparation and ending posture) (Zheng et al., 2016). Two professional coaches with over 5 years of Baduanjin teaching experience at the Fujian University of Traditional Chinese Medicine were employed to guide participants’ training.

In the brisk walking group, professional coaches were employed to guide participants’ training. The intensity of the exercise was controlled by maintaining participants’ heart rates at 55–75% of their heart rate reserve via the Polar Heart Rate Monitor.

Subjects in the healthy education control group were required to maintain their original physical activity levels and did not receive any specific exercise interventions except for the health education.

### Behavioral Outcome

The Chinese version of the Montreal Cognitive Assessment (MoCA-Chinese Beijing version) scale was selected as our primary clinical outcome measure to assess global cognitive function. MoCA is a brief cognitive screening instrument that was created and validated to detect MCI (about 10-min). There are 8 items (visuospatial/executive functions, naming, verbal memory registration and learning, attention, abstraction, 5-min delayed verbal memory, and orientation) with a total score of 0-30 (a higher score equates to better function). The MoCA-Chinese Beijing version demonstrated an excellent sensitivity of 90.4% and a fair specificity (31.3%) when the cut-off score was recommended 26 (Yu et al., 2012; Zheng et al., 2016). The MoCA was measured at baseline and within one week after the 24-week intervention for all participants.

### Functional and Structural MRI Data Acquisition

The fMRI data was acquired on a 3.0-T GE scanner (General Electric, Milwaukee, WI, United States) with an eight-channel phased array head coil at baseline and at the end of the intervention. Resting state functional MRI data was acquired with the following parameters: TR = 2100 ms, TE = 30 ms, flip angle = 90°, voxel size = 3.125 mm × 3.125 mm × 3.6 mm, 42 axial slices, field of view (FOV) = 200 mm × 200 mm, time pointes = 160. High-resolution structural images (MPRAGE) were acquired with the parameters of 7° flip angle, voxel size: 1 × 1 × 1 mm^3^, 240 mm FOV, and 164 slices. Subjects were asked to stay awake and remain motionless during the scan with their eyes closed and ears plugged.

### Data Analysis and Statistics

#### Behavioral Data Analysis

One-way ANOVA and Chi-square tests were applied to compare the baseline characteristics of subjects, and ANCOVA was applied to compare changes (post-treatment minus pre-treatment) in MoCA scores across all groups, adjusted for age (years), gender, education, and baseline MoCA scores using IBM SPSS Statistics software (Dimitrov and Rumrill, 2003).

#### Seed-to-Voxel Functional Connectivity Correlational Analysis

Similar to our previous study (Liu et al., 2019a, c; Tao et al., 2019), the seed-to-voxel functional connectivity correlational analyses were performed using a standard pipeline in a functional connectivity toolbox (CONN^1^) in MATLAB. The seeds (regions of interest) of bilateral LC and VTA (left and right separately) used in the present study were applied based on previous studies. The left LC was defined as 4 × 6 × 10 mm centered at MNI coordinates −5,−34,−21, and the right LC was defined as 4 × 6 × 10 mm centered at MNI coordinates 7,−34,−21 (Naidich et al., 2009; Bär et al., 2016). The VTA was defined as a 4 mm radius sphere (MNI coordinates left VTA: −4,−15,−9; right VTA: 5,−14,−8) (Adcock et al., 2006). The images were slice-time corrected, realigned, coregistered to subjects’ respective structural images, normalized, and smoothed with a 4 mm full width half maximum (FWHM) kernel. Then, segmentation of gray matter, white matter, and cerebrospinal fluid (CSF) areas for the removal of temporal confounding factors was employed. Band-pass filtering was performed with a frequency window of 0.01 to 0.089 Hz.

To eliminate correlations caused by head motion and artifacts, we identified outlier time points in the motion parameters and global signal intensity using ART^2^. We treated images as outliers if the composite movement from a preceding image exceeded 0.5 mm or if the global mean intensity was greater than 3 standard deviations from the mean image intensity (Wang Z. et al., 2017). The temporal time series of the head motion matrix of outliers were put into the first-level analysis as covariates (Whitfield and Nieto, 2012). A threshold of *p* < 0.005 uncorrected in voxel level and *p* < 0.05 FDR corrected at cluster level was used for group analysis. Due to the important role of the amygdala in the modulation of emotion, stress, and mind-body intervention (Tang et al., 2015) and the small size of the area, we pre-defined the bilateral amygdala as a region of interest (ROI). For the ROI (as defined by AAL brain atlas), a threshold of voxel-wise *p* < 0.005 was used in data analysis. Monte Carlo simulations using the 3dFWHMx and 3dClustSim [AFNI^3^ released in July 2017] were applied to correct for multiple comparisons (Gollub et al., 2018).

#### Exploratory Spectral Dynamic Causal Modeling Analysis

We found the overlapped brain regions in the right ACC and right insula among the rsFC of the right LC and left VTA (see results). To further investigate the causal interaction underlying the four brain regions (overlapped right ACC, overlapped right insula, ROIs of right LC and left VTA) during intervention, we performed the effective connectivity analysis using dynamic causal modeling (Sharaev et al., 2016; Li et al., 2017). The Spectral DCM analysis was performed using DCM12 (Wellcome Trust Centre for Neuroimaging, London, United Kingdom) implemented in SPM12. We specified two connectivity models: one model including the right LC, right ACC, and right insula (named the “right LC model”), and the other model including the left VTA, right ACC, and right insula (named the “left VTA model”). Four different sub-models were specified for each model: one fully connected model with bi-directional connections between all pairs of ROIs and three models where different regions predominantly affected the others (Sharaev et al., 2016; Figures 3A1,B1). In addition, to further investigate whether there were causal interactions between the left VTA and right LC, we defined a connectivity model that included all four brain masks (named the “right LC and left VTA model”). This model included five different sub-models: one fully connected model and four models where different regions predominantly affected the others (Supplementary Figure 1A). In this study, we assumed that all participants in the same conditions (i.e., pre-Baduanjin, post-Baduanjin, pre-walking, post-walking, pre-control, post-control) used the same model (the wining model).

Time series of the four brain masks of each subject (pre- and post-treatment separately) were extracted via a General Linear Model (GLM). The 6-rigid body realignment parameters (six head motion parameters) and the signals of cerebrospinal fluid and white matter were used as constant regresses in the GLM. Random Effects Bayesian model selection (RFX-BMS) was performed to determine the best model in each condition, considering both accuracy and complexity (Stephan et al., 2009). Repeated measures analysis was used to investigate the group and time interaction of the different connectivity parameters for the “wining” model.

#### Exploratory Region of Interest Voxel-Based Morphometry (VBM) Analysis

To investigate whether there were structural changes (gray matter volume, GMV) in the brain regions that showed significant group differences (Baduanjin vs. Control) and overlap between the rsFC of the right LC and left VTA (i.e., right amygdala, right ACC, and right insula; see Results for more information), we applied a region of interest VBM analysis (Andrew et al., 2018; Liu et al., 2019a, b; Tao et al., 2019) in SPM12. The data preprocessing for VBM analysis was similar to our previous study (Tao et al., 2017c). The T1 images of all participants were segmented into gray matter, white matter, and cerebrospinal fluid. Then, a group specific template was created after the images were normalized using the high dimensional DARTEL algorithm. Spatial smoothing was conducted with a 6 mm FWHM after the template was normalized into the standard Montreal Neurological Institute (MNI) space. A factorial design module with two factors (i.e., groups with three levels and time with two levels) was applied to explore the group differences. Age and gender were also included in the model as covariates. An absolute threshold of 0.1 was used for masking (Tao et al., 2017c). Total intracranial volume was obtained by summing up the overall volumes of gray matter, white matter, and cerebrospinal fluid. Then, we extracted the average GMV values of all voxels with the overlapping brain masks (i.e., right ACC, right amygdala, and right insula) in the three groups derived from functional connectivity analysis. ANCOVA was applied to compare changes of the three groups (post-treatment minus pre-treatment) with age and gender as covariates (Dimitrov and Rumrill, 2003).

## Results

Sixty-nine MCI patients completed the baseline behavioral test and MRI scan, and 57 subjects finished the study and were included in data analysis (Baduanjin group *n* = 20, brisk walking group *n* = 17, and health education control group *n* = 20). Three subjects dropped out of the Baduanjin group (one subject withdrew voluntarily, and two were unwilling to participate in the second MRI scan). Six subjects dropped out of the brisk walking group (one withdrew voluntarily, one was unwilling to participate in the second MoCA test, one was lost to follow-up, and three had poor-quality MRI data). Three subjects dropped out of the control group (one was lost to follow-up, and two were unwilling to participate in the second MoCA test). No subject reported taking additional pharmacological treatment during the training. The attendance rates (mean ± SD) for the Baduanjin and brisk walking groups were 0.842 ± 0.100 and 0.838 ± 0.116, respectively, and there were no significant differences between the two exercise groups in terms of attendance rate (*p* = 0.927).

### Behavioral Results

Baseline characteristics and MoCA scores of participants are listed in Table 1. There were no significant differences in age, gender, education, score on the Geriatric depression scale (GDS), or MoCA scores among the three groups at baseline (three-group comparison *p*-Value as follows: Age: *p* = 0.395; Gender: *p* = 0.563; Education: *p* = 0.675; GDS: *p* = 0.12; pre-MoCA: *p* = 0.141).

**TABLE 1:** Demographics of study participants and clinical outcome results.

	**Baduanjin (*n* = 20)**	**Walking (*n* = 17)**	**Control (*n* = 20)**	***F* or Chi-square value**	***P*-value**
Age†(mean(SD))	66.17(4.17)	64.32(2.60)	65.97(5.66)	0.945	0.395
Gender‡(male/female)	5/15	7/10	6/14	1.148	0.563
Education‡ (1/2/3/4)	3/6/6/5	2/5/9/1	3/4/8/5	4.016	0.675
Geriatric depression scale (GDS) †(mean(SD))	6.05(2.84)	4.18(2.65)	5.50(2.76)	2.204	0.120
Pre-MoCA†(mean(SD))	22.45(2.16)	21.47(2.27)	21.00(2.36)	2.035	0.141
MoCA change (mean(SD))	2.10(2.25)	0.88(1.96)	1.10(1.48)	4.311	0.019

*†*p*-Values were calculated with one-way analysis of variance, ‡*p*-Values were calculated with a chi-square test. Education 1: primary school; 2: middle school; 3: high school; 4: college and above. Baduanjin: Baduanjin group; Walking: brisk walking group; Control: non-exercise group; MoCA change: post-treatment MoCA score minus pre-treatment MoCA.*

ANCOVA showed a significant difference in MoCA score changes (post-treatment minus pre-treatment) among the three groups (*F*_2_,_50_ = 4.311; *p* = 0.019). *Post hoc* analysis showed that the Baduanjin group had a significant increase in MoCA scores compared to the health education control group (*p* = 0.05) and the brisk walking group (*p* = 0.037) after Sidak correction. There was no significant difference between the brisk walking group and the control group (*p* = 0.989, Table 1).

### Seed-to-Voxel rsFC Results

In present study, we used T2 imaging data to check whether the participants have underlying brain diseases, such as brain tumors or lesions. No obvious brain abnormalities were detected. No subject was excluded due to the head movement in the study.

#### Seed-to-Voxel rsFC Analysis Using the LC as the Seed

**FIGURE 1:**
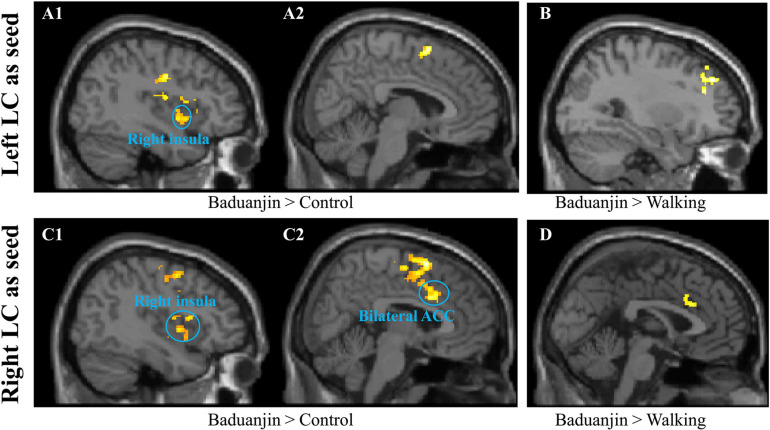
Resting state functional connectivity results using the LC as the seed. **(A1,A2,B)**: using the left LC as the seed. **(A1,A2)**, Baduanjin > Control; **(B)** Baduanjin > Brisk Walking. **(C1,C2,D)**, using the right LC as the seed. **(C1,C2)**, Baduanjin > Control; **(D)** Baduanjin > Brisk Walking.

Seed-to-voxel rsFC analysis using the left LC as the seed showed increased rsFC in the bilateral temporoparietal junction (TPJ), right insula, inferior frontal gyrus, supplemental motor area, and postcentral gyrus in the Baduanjin group compared to the control group. The analysis also showed increased rsFC in the right dorsolateral prefrontal cortex and decreased rsFC in the bilateral cerebellum exterior in the Baduanjin group compared to the brisk walking group. There was no other significant group difference at the threshold we set (Figures 1A1,A2,B and Table 2).

**TABLE 2:** Resting state functional connectivity results using LC and VTA as seeds.

**Seed**	**Contrast**	**Cluster**	***T*-value**	***Z*-value**	**MNI coordinate**			**Brain region**
					**x**	**y**	**z**	
Left LC	Baduanjin > Control	285	4.39	3.90	52	0	6	R insula
		113	4.61	4.06	52	−32	16	R TPJ
		130	3.94	3.57	−26	−36	40	L TPJ
		128	5.31	4.53	34	24	8	R inferior frontal
		135	5.29	4.52	8	12	66	R supplemental motor area
		120	4.37	3.89	48	14	14	R inferior frontal
		180	4.31	3.84	40	−8	36	R postcentral gyrus
	Control > Baduanjin	No brain region above threshold						
	Baduanjin > Walking	127	4.26	3.78	32	36	26	R DLPFC
	Walking > Baduanjin	152	4.34	3.83	−2	−62	−46	Bilateral Cerebellum exterior
	Walking > Control	No brain region above threshold						
	Control > Walking	No brain region above threshold						
Right LC	Baduanjin > Control	294	4.58	4.04	38	10	12	R insula
		21	3.58	3.29	20	4	−30	R amygdala
		198	4.82	4.2	52	−38	18	R TPJ
		643	5.81	4.85	4	8	64	R supplemental motor area
			3.77	3.44	6	12	38	Bilateral ACC
		423	5.24	4.48	−62	−2	6	L precentral gyrus
		107	4.86	4.23	24	−26	68	R precentral gyrus
		119	4.08	3.678	62	4	−2	R superior temporal gyrus
		345	4.59	4.05	44	2	54	R precentral gyrus
	Control > Baduanjin	No brain region above threshold						
	Baduanjin > Walking	125	4.63	4.04	6	14	32	Right ACC
	Walking > Baduanjin							
		96	5.86	4.82	4	−8	60	R supplement motor cortex
		91	4.82	4.17	42	−80	2	R inferior occipital cortex
		94	3.04	2.83	32	−22	60	R precentral cortex
		95	3.03	2.83	−60	−10	34	L postcentral cortex
		91	4.44	3.90	−30	−30	56	L precentral cortex
		189	4.08	3.64	58	−6	22	R pre & postcentral cortex
		99	4.04	3.62	−28	−94	14	L middle occipital cortex
		116	6.23	5.03	38	−60	−56	R cerebellum
	Walking > Control	No brain region above threshold						
	Control > Walking	No brain region above threshold						
Left VTA	Baduanjin > Control	527	3.77	3.43	40	2	2	R anterior insula
			3.50	3.22	20	2	−30	R amygdala
			5.20	4.46	22	12	0	R putamen
			5.15	4.43	−16	14	−2	L putamen/caudate
			4.24	3.80	22	16	−24	R orbital frontal gyrus
			4.14	3.72	10	14	0	R caudate
			3.99	3.61	−8	8	−8	L nucleus accumbent
		107	4.70	4.12	−28	24	2	L anterior insula
		186	4.97	4.31	54	−6	30	R postcentral gyrus
		107	4.05	3.65	−26	−56	56	L superior parietal gyrus
	Control > Baduanjin	130	5.00	4.33	−20	−54	22	L PCC
	Baduanjin > Walking	173	5.27	4.46	2	−4	36	Bilateral ACC
	Walking > Baduanjin	No brain region above threshold						
	Walking > Control	No brain region above threshold						
	Control > Walking	126	4.69	4.08	−4	−60	56	L precuneus
Right VTA	Baduanjin > Control	No brain region above threshold						
	Control > Baduanjin	No brain region above threshold						
	Baduanjin > Walking	154	6.59	5.23	28	−42	44	R TPJ
	Walking > Baduanjin	No brain region above threshold						
	Walking > Control	No brain region above threshold						
	Control > Walking	278	4.36	3.85	−4	−56	60	Bilateral precuneus

*ACC: anterior cingulate cortex; PCC: posterior cingulate cortex; DLPFC: dorsolateral prefrontal cortex; TPJ: temporoparietal joint; L: left; R: right.*

Seed-to-voxel rsFC analysis using the right LC as the seed showed that Baduanjin group is associated with increased rsFC in the bilateral ACC, bilateral precentral gyrus, right insula, amygdala, TPJ, supplemental motor area, and superior temporal gyrus compared to the control group, as well as increased rsFC in the right ACC compared to the brisk walking group. Compared to the Baduanjin group, there was significantly increased rsFC in the right supplemental motor cortex, inferior occipital cortex, bilateral precentral & postcentral cortex, left middle occipital cortex, and right cerebellum in the brisk walking group. There was no other significant group difference at the threshold we set (Figures 1C1,C2,D and Table 2).

Interestingly, we found an overlapping increased right LC-right ACC rsFC in the Baduanjin group compared to the control and brisk walking groups. We also found that both the left and right LC are associated with increased rsFC with the right insula in the Baduanjin group compared to the control group. We extracted the average *z*-Values of the two rsFCs associated with the right LC (rsFC of right ACC-right LC and rsFC of right insula-right LC) in the three groups after treatment and performed a multiple regression analysis including age and gender as covariates. We found a significant positive association between rsFC *z*-Values at the right ACC and right insula (right ACC: *r* = 0.265, *p* = 0.046; right insula: *r* = 0.277, *p* = 0.037) and corresponding MoCA scores after FDR correlation across all subjects in three groups.

#### Seed-to-Voxel rsFC Analysis Using the VTA as the Seed

Seed-to-voxel analysis using the left VTA as the seed showed significantly increased rsFC in the bilateral anterior insula, putamen, caudate; right amygdala, orbital frontal gyrus, postcentral gyrus; and left nucleus accumbent and superior parietal gyrus in the Baduanjin group compared to the control group. Analysis also showed significantly decreased rsFC in the left posterior cingulate cortex in the Baduanjin group compared to the control group and increased rsFC in the bilateral ACC compared to the brisk walking group. We also found significantly decreased rsFC in the left precuneus in the brisk walking group compared to the control group. We did not find any other significant group differences above the threshold we set (Table 2).

Seed-to-voxel analysis using the right VTA as the seed showed an increased rsFC in the right TPJ in the Baduanjin group compared to the brisk walking group. There was also a decreased rsFC in the bilateral precuneus in the brisk walking group compared to the control group. No other significant group difference was found at the threshold we set (Table 2).

**FIGURE 2:**
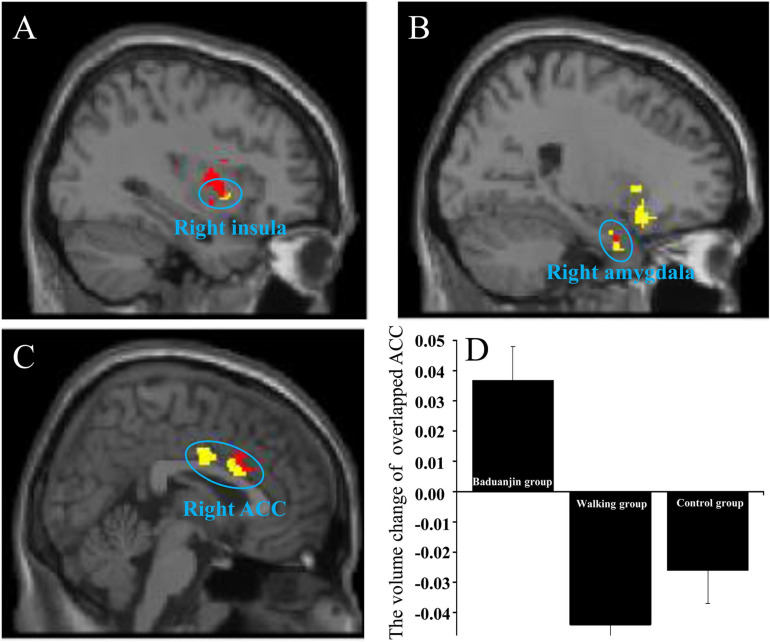
The increased overlapping rsFC in the Baduanjin group between the right LC and left VTA compared to the control group/walking group and the VBM analysis of the overlapping brain region. **(A)** the overlapping brain region in the right insula; **(B)** the overlapping brain region in the right amygdala; **(C)** the overlapping brain region in the right ACC; Red, using the right LC as the seed; yellow, using the left VTA as the seed; **(D)** the ROI VBM analysis in the right ACC among the three groups.

We found that both the right LC and left VTA are associated with increased rsFC with the right insula and right amygdala in the Baduanjin group as compared to the control group (the rsFCs of right insula-right LC/left VTA; the rsFCs of right amygdala-right LC/left VTA). We also found an overlapping brain region in the right ACC in the two rsFCs analyses (i.e., the rsFC of right LC-right ACC and the rsFC of left VTA-bilateral ACC) in the Baduanijn group compared to the brisk walking group (Figures 2A-C).

### Spectral Dynamic Causal Modeling Results

**FIGURE 3:**
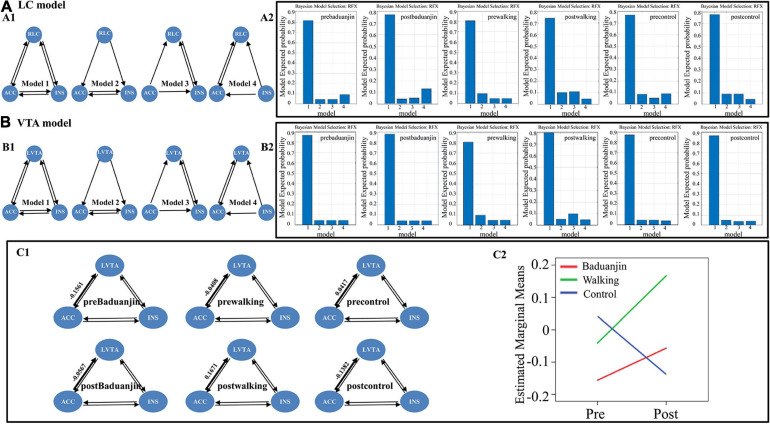
The “right LC model” and “left VTA model” setup, the winning sub-model at the condition level in the “right LC model” and “left VTA model,” and the group and time interaction of right ACC to left VTA effective connectivity. **(A)** The “right LC model” and the winning sub-model at the condition level in the “right LC model”. **(A1)** right LC model setup: model 1-4, sub-model of the “right LC model,” model 1-full connective model, model 2- right LC predominantly affected the others, model 3- right ACC predominantly affected the others, model 4-right insula predominantly affected the others; **(A2)** the winning sub-model of the “right LC model” in each condition (i.e., pre-Baduanjin, post-Baduanjin, pre-walking, post-walking, pre-control, post-control); **(B)** The “left VTA model” and the winning sub-model at the condition level in the “left VTA model”. **(B1)** Left VTA model setup: model 1-4, sub-model of “left VTA model,” model 1-full connective model, model 2-left VTA predominantly affected the others, model 3-rigth ACC predominantly affected the others, model 4-right insula predominantly affected the others; **(B2)** the winning sub-model of the “left VTA model” in each condition (i.e., pre-Baduanjin, post-Baduanjin, pre-walking, post-walking, pre-control, post-control). **(C)** Mean connection strengths and the group and time interaction of right ACC to left VTA effective connectivity. **(C1)** Mean connection strengths for the effective connectivity of right ACC to left VTA. **(C2)** The group and time interaction for the effective connectivity of right ACC to left VTA. RLC: right locus coeruleus; LVTA: left ventral tegmental area; ACC: right overlapped anterior cingulate cortex; INS: right overlapped insula.

Random Effects Bayesian model analysis showed that the fully connected model was the best model at each condition (i.e., pre-Baduanjin, post-Baduanjin, pre-walking, post-walking, pre-control, post-control) in the “right LC model,” “left VTA model” (Figures 3A,B), and “right LC and left VTA model” (Supplementary Figure 1B, see the Supplementary Materials for details).

We then performed the repeated measures analysis in each pair of endogenous connectivity (DCM.Ep.A) of the full model. No significant group and time interaction was found in the “right LC model” and “right LC and left VTA” model in all effective connectivity (see Table 3 and Supplementary Tables 1, 2; for the right LC model, the group^∗^time interaction *P*-value).

**TABLE 3:** The group and time interaction in the effective connectivity.

**Effective connectivity**	**Time main effect *P*-value**	Time*Group interaction *P*-value
**Right LC model**		
LC→ACC	0.050	0.334
LC→INS	0.817	0.732
ACC→LC	0.881	0.066
ACC→INS	0.246	0.515
INS→LC	0.455	0.588
INS→ACC	0.071	0.341
**Left VTA model**		
VTA→ACC	0.669	0.444
VTA→INS	0.913	0.404
ACC→VTA	0.502	0.041
ACC→INS	0.518	0.819
INS→VTA	0.084	0.291
INS→ACC	0.630	0.880

*LC: ROI of right locus coeruleus; VTA: ROI of left ventral tegmental area; ACC: right overlapped anterior cingulate cortex; INS: right overlapped insula.*

We found increased effective connectivity from the right ACC to the left VTA in the Baduanjin and brisk walking groups and decreased effective connectivity in the control group after intervention, with significant group and time interactions (*p* = 0.041) in the “left VTA model.” No other significant effective connectivity group and time interaction was found in the “left VTA model” (Figures 3C1, C2, Table 3, and Supplementary Table 1).

### Region of Interest Voxel-Based Morphometry (VBM) Analysis

We compared VBM changes (post-treatment minus pre-treatment) at the right ACC, right insula, and right amygdala after different treatments and found a significant group difference in right ACC GMV (*F*_2_,_50_ = 14.076; *p* < 0.001). *Post hoc* analysis revealed increased GMV in the right ACC of the Baduanjin group compared to the control group (*p* < 0.001) and brisk walking group (*p* < 0.001), but no significant group difference between the brisk walking and control groups (*p* = 0.621) after Sidak correction (Figure 2D). No significant GMV difference was found in the right insula and right amygdala among groups (*p* > 0.05).

## Discussion

In the present study, we investigated the modulation effects of 6 months of Baduanjin on LC and VTA system in patients with MCI. We found that Baduanjin can significantly modulate the rsFC of the LC and VTA and increase gray matter volume of the right ACC compared to brisk walking and health education. In addition, we found a significant group and time interaction in the effective connectivity from right ACC to left VTA. Our results support our research hypothesis that Baduanjin can modulate intrinsic functional connectivity of norepinephrine (LC) and dopamine (VTA) systems.

Our findings that Baduanjin can significantly improve the cognitive function in patients with MCI are consistent with findings from a previous study that endorsed the treatment effects of mind-body exercise on MCI (Sungkarat et al., 2018), as well as our previous studies demonstrating that Baduanjin can prevent memory decline in healthy elders (Tao et al., 2017a,b,c). Interestingly, we did not find significant cognitive function improvement in the brisk walking group compared to the health education group. Some studies have suggested a positive effect of physical exercise on improving cognitive function or preventing cognitive decline (Baker et al., 2010; Brasure et al., 2018). However, a recent clinical study showed that intense aerobic exercise and strength exercise training resulted in no significant group differences in slowing cognitive impairment in people with mild to moderate dementia (Sarah et al., 2018). Further studies are needed to confirm our results.

We found that, compared to the brisk walking group, Baduanijn exercise significantly increased the rsFC between the right ACC and left VTA/right LC. The gray matter volume of the right ACC was significantly increased after 6 months of Baduanjin exercise compared to the brisk walking and health education groups. These findings are consistent with previous studies indicating the role of the ACC in cognition as well as a previous study based on the same data set in which we found increased amplitude of low-frequency fluctuations values (a measurement of brain low-frequency oscillations) in the bilateral ACC in the Baduanjin group compared to the control group (Tao et al., 2019).

Previous studies have found that ACC activation is involved in a number of cognitive processes, including decision making, model updating, and outcome-related activity (Kolling et al., 2016). Animal studies have suggested that the ACC provides prominent direct input to the LC, and that the connectivity between the LC and ACC is involved in cognitive processes like reinforcement learning (Aston-Jones and Cohen, 2005). The ACC may also be able to exert direct top-down control over the VTA (Thomas and David, 2017; Wang S. et al., 2017).

A previous study found that LC-NE and VTA-dopamine systems may interact synergistically to implement an auto-annealing reinforcement learning mechanism (Aston-Jones and Cohen, 2005). This finding aligns with our results, which showed overlapping rsFC in the ACC between the left LC and right VTA. Our results suggest that the ACC may play an important role in Baduanjin modulation by linking LC-NE and VTA- dopamine systems.

In addition, we found that the exercise groups had an increased effective connectivity from the right ACC to the left VTA and a marginally significant increase from the right ACC to the right LC (*p* = 0.066) compared to the control group (Table 3 and Supplementary Table 1). Effective connectivity analysis using DCM (Friston et al., 2003) can describe the causal relationships between brain regions in the functional MRI data (Friston and Penny, 2011; Sharaev et al., 2016). Specifically, effective connectivity can measure the influence that one neuronal system/brain region exerts on another (Sharaev et al., 2016). Recently, the method has been applied to investigate the modulation effect of mind-body intervention (Kong et al., 2021). Previous studies have suggested that the ACC has reciprocal anatomical connections with both the LC and VTA (Leichnetz and Astruc, 1976; Carmichael and Price, 1995). We found that the ACC has an increased causal influence on the VTA and LC (marginal significant) after exercise. This suggests that the ACC may play a major role in the modulation effect of exercise in patients with MCI.

The insula is a key member of the salience network and plays a role in regulating mental allocation (Ullsperger et al., 2010). Previous studies have suggested that anatomical connection between the LC and anterior insula may be associated with the processing of unexpected events (Dayan and Yu, 2006; Ullsperger et al., 2010). Hämmerer et al. (2018) found that older adults perform worse in a salient stimulation memory task than young adults. In addition, the insula is a dopaminergic region that functions to process stimulus salience and motivation, integrating information about task salience and regulating VTA excitability in response to reward (Rigoli et al., 2016). The above evidence suggests that the regulatory effect of the insula in cognitive function (especially in memory processing) of elders may be associated with the VTA and LC. In addition, studies have suggested that the structure and function of the insula can be significantly modulated by mindfulness training (Hodie lzel et al., 2008; Ives-Deliperi et al., 2011). We speculate that the increased rsFC between the VTA/LC and insula after Baduanjin training may represent the potential modulation of interoceptive awareness skills and salience event processing.

We also found an overlapping increased rsFC in the amygdala between the VTA and LC after Baduanjin exercise. Literature suggests that the amygdala plays a key role in the interaction of emotion and cognition (Phelps, 2006) and has a direct anatomical connection with the VTA-dopaminergic system (Thomas et al., 2018) and LC-noradrenergic system (Mohammed et al., 2016). A series of studies has suggested that stress affects the structure of the amygdala (Mitra et al., 2005; McEwen et al., 2016). Thus, Baduanjin may relieve stress and negative emotion (a key risk factor for the development of MCI) by modulating the interaction between the LC and VTA in the amygdala and further improving cognitive function.

We found increased rsFC of the LC-TPJ and VTA-TPJ in the Baduanjin group compared to both the control group and brisk walking group. The TPJ plays a key role in memory processing and social cognition (Carter and Huettel, 2013). Both the LC and TPJ are involved in executive and attentional processes (Laureiro-Martínez et al., 2015), and the functional connectivity between the TPJ and reward-related regions (such as the VTA) is involved in episodic encoding (Sugimoto et al., 2016). These results indicate that the TPJ may also be involved in the modulation effect of Baduanjin in cognition.

There are several limitations to our study. First, we did not evaluate levels of norepinephrine and dopamine directly. Further studies are needed to assess these neurotransmitter levels with neuroimaging techniques. Additionally, our sample size in this study was relatively small. Studies with larger sample sizes should be conducted to confirm our findings. Finally, we only measured global cognitive function via MoCA, and further research should be conducted to explore the treatment effects of Baduanjin on cognitive functions like memory and attention in patients with MCI.

## Conclusion

In summary, we found that 6 months of Baduanjin exercise can significantly improve cognitive function compared to brisk walking and health education. In addition, Baduanjin can yield increases in LC and VTA rsFC with the ACC, insula, amygdala, and TPJ, increases in the effective connection of the ACC to LC/VTA, and increases in the gray matter volume of the ACC. Our findings suggest that Baduanjin may improve cognitive function in patients with MCI by modulating brain function and structure associated with the norepinephrine (LC) and dopamine (VTA) systems.

## Data Availability Statement

The raw data supporting the conclusions of this article will be made available by the authors, without undue reservation.

## Ethics Statement

The studies involving human participants were reviewed and approved by The Medical Ethics Committee of the Second People’s Hospital of Fujian Province. The patients/participants provided their written informed consent to participate in this study. Written informed consent was obtained from the individual(s) for the publication of any potentially identifiable images or data included in this article.

## Author Contributions

GZ and LC designed the experimental. RX, ML, MH, and SL contributed to the data collection. JL, JK, and JT contributed to the data analysis. JL, JK, JT, XC, GW, and JP prepared the manuscript. All authors contributed to the article and approved the submitted version.

## Conflict of Interest

JK has a disclosure to report (holding equity in a MNT, and pending patents to develop a new brain stimulation device). The remaining authors declare that the research was conducted in the absence of any commercial or financial relationships that could be construed as a potential conflict of interest.

## Footnotes

1 http://www.nitrc.org/projects/conn

2 https://www.nitrc.org/projects/artifact_detect

3 https://afni.nimh.nih.gov

## Supplementary Material

Supplementary Figure 1 The “right LC and left VTA model” setup and the winning sub-model at the condition level in the “right LC and left VTA model.” **(A)** model 1-5, sub-model of the “right LC and left VTA model,” model 1-full connective model, model 2-left VTA predominantly affected the others, model 3-right ACC predominantly affected the others, model 4-right insula predominantly affected the others; model 5-right LC predominantly affected the others; **(B)** the winning sub-model at the condition level in the “right LC and left VTA model.” RLC: right locus coeruleus; LVTA: left ventral tegmental area; ACC: right overlapped anterior cingulate cortex; INS: right overlapped insula.

Supplementary Figure 2 The number of subjects in each condition in each sub-model in the “right LC model,” “left VTA model,” and “right LC and left VTA model.”

Supplementary Table 1 Mean and Standard deviations of connection strengths.

Supplementary Table 2 The group and time interaction in the effective connectivity (“right LC and left VTA” model).

## Abbreviations

MCI: mild cognitive impairment
MoCA: Montreal Cognitive Assessment
MRI: magnetic resonance imaging
LC: locus coeruleus
VTA: ventral tegmental area
rsFC: resting state functional connectivity
ACC: anterior cingulate cortex
GMV: gray matter volume
VBM: Voxel-Based Morphometry
TPJ: temporoparietal junction
DLPFC: dorsolateral prefrontal cortex.
