# Supplementary figures and images for: Mind-Body Exercise Modulates Locus Coeruleus and Ventral Tegmental Area Functional Connectivity in Individuals With Mild Cognitive Impairment
Source: Front Aging Neurosci. 2021 Jun 14;13:646807. doi: 10.3389/fnagi.2021.646807 (PMC8236862; doi:10.3389/fnagi.2021.646807)

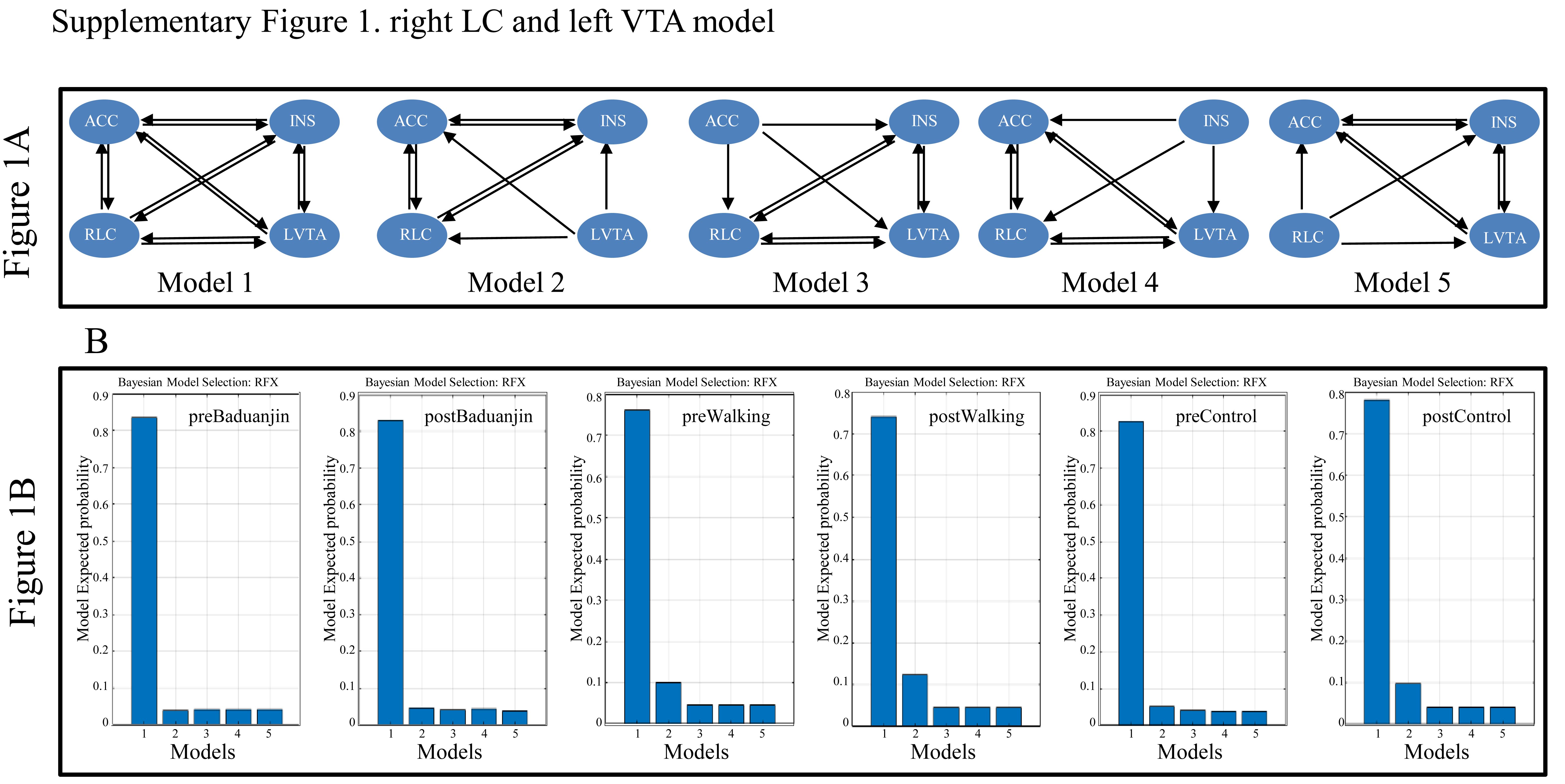

Supplement: Supplementary Figure 1 — The “right LC and left VTA model” setup and the winning sub-model at the condition level in the “right LC and left VTA model.” (A) model 1-5, sub-model of the “right LC and left VTA model,” model 1-full connective model, model 2-left VTA predominantly affected the others, model 3-right ACC predominantly affected the others, model 4-right insula predominantly affected the others; model 5-right LC predominantly affected the others; (B) the winning sub-model at the condition level in the “right LC and left VTA model.” RLC: right locus coeruleus; LVTA: left ventral tegmental area; ACC: right overlapped anterior cingulate cortex; INS: right overlapped insula. [file Image_1.jpeg]

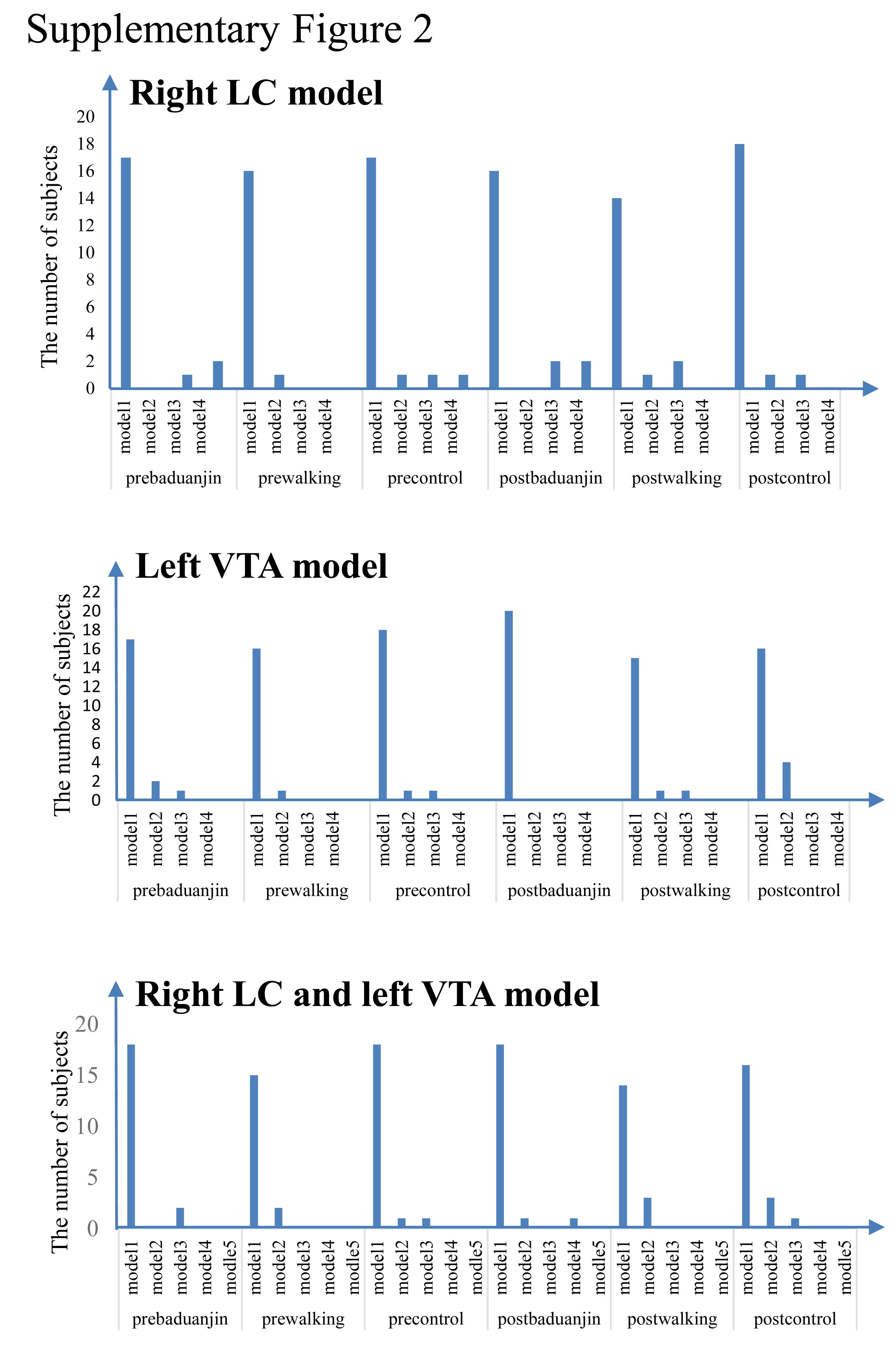

Supplement: Supplementary Figure 2 — The number of subjects in each condition in each sub-model in the “right LC model,” “left VTA model,” and “right LC and left VTA model.” [file Image_2.jpeg]
