# Supplementary material for: Mind-Body Exercise Modulates Locus Coeruleus and Ventral Tegmental Area Functional Connectivity in Individuals With Mild Cognitive Impairment
Source: Front Aging Neurosci. 2021 Jun 14;13:646807. doi: 10.3389/fnagi.2021.646807 (PMC8236862; doi:10.3389/fnagi.2021.646807)
Supplement: Supplementary Table 1 — Mean and Standard deviations of connection strengths. [file Table_1.doc]

**Supplementary table 1. Mean and Standard deviations of connection strengths**

| **Effective connectivity** | **Prebaduanjin**  **mean(SD)** | **Prewalking**  **mean(SD)** | **Precontrol**  **mean(SD)** | **Postbaduanjin**  **mean(SD)** | **Postwalking**  **mean(SD)** | **Postcontrol**  **mean(SD)** |
| --- | --- | --- | --- | --- | --- | --- |
| **LC** | | | | | | |
| LC→ACC | 0.026(0.451) | -0.197(0.615) | 0.120(0.439) | 0.165(0.492) | 0.142(0.424) | 0.149(0.486) |
| LC→INS | 0.088(0.461) | 0.002(0.562) | 0.179(0.495) | 0.153(0.398) | 0.064(0.587) | 0.108(0.475) |
| ACC→LC | 0.168(0.565) | 0.053(0.417) | 0.269(0.526) | 0.367(0.438) | 0.137(0.460) | 0.023(0.620) |
| ACC→INS | -0.004(0.525) | 0.218(0.616) | 0.132(0.527) | 0.229(0.469) | 0.301(0.499) | 0.125(0.509) |
| INS→LC | 0.123(0.518) | 0.333(0.362) | 0.208(0.458) | 0.072(0.572) | 0.129(0.446) | 0.247(0.557) |
| INS→ACC | 0.261(0.657) | 0484(0.444) | 0.364(0.575) | 0.189(0.635) | 0.036(0.554) | 0.264(0.497) |
| **VTA** | | | | | | |
| VTA→ACC | 0.161(0.480) | 0.346(0.900) | 0.349(0.711) | 0.280(0.711) | 0.083(0.646) | 0.337(0.560) |
| VTA→INS | 0.253(0.632) | 0.258(0.556) | 0.188(0.677) | 0.270(0.538) | 0.027(0.753) | 0.362(0.681) |
| ACC→VTA | -0.156(0.357) | -0.041(0.324) | 0.042(0.465) | -0.057(0.389) | 0.167(0.286) | -0.138(0.386) |
| ACC→INS | 0.058(0.407) | 0.467(0.449) | 0.226(0.444) | 0.074(0.546) | 0.379(0.309) | 0.160(0.524) |
| INS→VTA | 0.066(0.354) | 0.069(0.365) | -0.106(0.412) | 0.221(0.349) | 0.039(0.320) | 0.123(0.367) |
| INS→ACC | 0.489(0.399) | 0.010(0.502) | 0.300(0.416) | 0.475(0.406) | 0.148(0.468) | 0.377(0.524) |
| **LC and VTA** | | | | | | |
| VTA→ACC | 0.104(0.588) | 0.410(0.535) | 0.239(0.462) | 0.211(0.600) | 0.056(0.673) | 0.335(0.521) |
| VTA→INS | 0.153(0.491) | 0.363(0.346) | 0.326(0.390) | 0.216(0.511) | 0.103(0.754) | 0.289(0.417) |
| VTA→LC | 0.249(0.438) | 0.536(0.355) | 0.414(0.475) | 0.435(0.493) | 0.520(0.480) | 0.268(0.411) |
| ACC→VTA | -0.051(0.330) | -0.077(0.375) | -0.081(0.324) | 0.076(0.361) | 0.101(0.289) | -0.131(0.354) |
| ACC→INS | 0.094(0.361) | 0.295(0.397) | 0.099(0.390) | 0.085(0.458) | 0.195(0.403) | -0.003(0.460) |
| ACC→LC | 0.170(0.376) | 0.026(0.323) | 0.032(0.519) | 0.053(0.465) | 0.113(0.355) | 0.173(0.523) |
| INS→VTA | 0.047(0.317) | 0.007(0.215) | -0.005(0.307) | -0.029(0.450) | 0.036(0.319) | 0.014(0.351) |
| INS→ACC | 0.336(0.415) | 0.190(0.460) | 0.343(0.383) | 0.156(0.373) | 0.226(0.492) | 0.280(0.472) |
| INS→LC | 0.128(0.378) | 0.201(0.289) | 0.237(0.442) | 0.133(0.459) | 0.042(0.366) | 0.122(0.471) |
| LC→VTA | -0.005(0.336) | 0.080(0.298) | 0.004(0.248) | -0.002(0.225) | -0.045(0.289) | 0.060(0.383) |
| LC→ACC | -0.025(0.364) | -0.060(0.489) | 0.050(0.408) | 0.191(0.372) | 0.048(0.254) | 0.040(0.368) |
| LC→INS | 0.018(0.338) | -0.059(0.380) | 0.052(0.404) | 0.124(0.428) | -0.006(0.459) | 0.165(0.489) |

LC: ROI of right locus coeruleus; VTA: ROI of left ventral tegmental area; ACC: right overlapped anterior cingulate cortex; INS: right overlapped insula.
