# Supplementary material for: Mind-Body Exercise Modulates Locus Coeruleus and Ventral Tegmental Area Functional Connectivity in Individuals With Mild Cognitive Impairment
Source: Front Aging Neurosci. 2021 Jun 14;13:646807. doi: 10.3389/fnagi.2021.646807 (PMC8236862; doi:10.3389/fnagi.2021.646807)
Supplement: Supplementary Table 2 — The group and time interaction in the effective connectivity (“right LC and left VTA” model). [file Table_2.doc]

**Supplementary Table 2. The group and time interaction in the effective connectivity** (“right LC and left VTA” model)

| **Effective connectivity** | **Time main effect P value** | **Time*Group interaction P value** |
| --- | --- | --- |
| VTA→ACC | 0.636 | 0.147 |
| VTA→INS | 0.401 | 0.366 |
| VTA→LC | 0.926 | 0.267 |
| ACC→VTA | 0.174 | 0.292 |
| ACC→INS | 0.261 | 0.780 |
| ACC→LC | 0.592 | 0.266 |
| INS→VTA | 0.884 | 0.762 |
| INS→ACC | 0.415 | 0.587 |
| INS→LC | 0.212 | 0.620 |
| LC→VTA | 0.632 | 0.338 |
| LC→ACC | 0.110 | 0.345 |
| LC→INS | 0.243 | 0.945 |

LC: ROI of right locus coeruleus; VTA: ROI of left ventral tegmental area; ACC: right overlapped anterior cingulate cortex; INS: right overlapped insula.
